# A computer-based quantitative systems pharmacology model of negative symptoms in schizophrenia: exploring glycine modulation of excitation-inhibition balance

**DOI:** 10.3389/fphar.2014.00229

**Published:** 2014-10-21

**Authors:** Athan Spiros, Patrick Roberts, Hugo Geerts

**Affiliations:** ^1^Computational Neuropharmacology, In Silico Biosciences, Inc.Berwyn, PA, USA; ^2^Department of Biomedical Engineering, Oregon Health and Science UniversityPortland, OR, USA; ^3^Department of Laboratory Pathology, Perelman School of Medicine, University of PennsylvaniaPhiladelphia, PA, USA

**Keywords:** schizophrenia, negative symptoms, glycine, computer model, dose-response relationship, drug

## Abstract

Although many antipsychotics can reasonably control positive symptoms in schizophrenia, patients' return to society is often hindered by negative symptoms and cognitive deficits. As an alternative to animal rodent models that are often not very predictive for the clinical situation, we developed a new computer-based mechanistic modeling approach. This Quantitative Systems Pharmacology approach combines preclinical basic neurophysiology of a biophysically realistic neuronal ventromedial cortical-ventral striatal network identified from human imaging studies that are associated with negative symptoms. Calibration of a few biological coupling parameters using a retrospective clinical database of 34 drug-dose combinations resulted in correlation coefficients greater than 0.60, while a robust quantitative prediction of a number of independent trials was observed. We then simulated the effect of glycine modulation on the anticipated clinical outcomes. The quantitative biochemistry of glycine interaction with the different NMDA-NR_2_ subunits, neurodevelopmental trajectory of the NMDA-NR_2B_ in the human schizophrenia pathology, their specific localization on excitatory vs. inhibitory interneurons and the electrogenic nature of the glycine transporter resulted in an inverse U-shape dose-response with an optimum in the low micromolar glycine concentration. Quantitative systems pharmacology based computer modeling of complex humanized brain circuits is a powerful alternative approach to explain the non-monotonic dose-response observed in past clinical trial outcomes with sarcosine, D-cycloserine, glycine, or D-serine or with glycine transporter inhibitors. In general it can be helpful to better understand the human neurophysiology of negative symptoms, especially with targets that show non-monotonic dose-responses.

## Introduction

Negative symptoms in schizophrenia are a major cause of functional deficit for patients wanting to return to professional life. While many of the approved antipsychotics can control the positive symptoms, negative symptom dysfunction is often not addressed properly by drug therapy alone (Rosenbaum et al., 2012). In addition, there are species differences for animal models that have large ramifications for drug development in schizophrenia (Peleg-Raibstein et al., 2012) and consequently psychiatric disorders have one of the lowest probabilities of clinical success, close to 7% (Hay et al., 2014). Because of these limitations, companies are de-emphasizing psychiatric diseases (Hyman, 2014), suggesting a need for completely novel technologies.

Negative symptoms can be divided in two moderately correlated factors (Horan et al., 2011): experiential impairments (diminished motivation and enjoyment of social, vocational, and recreational activities) and expressive impairments (diminished non-verbal and verbal communication). Experiential impairments are best represented by avolition and anhedonia, while expressive impairments are related to flat affect. Both these dimensions play an important role in the clinical phenotype.

Glutamate modulation through increased glycine mediated stimulation of the NMDA-R has been proposed as a strategy for addressing negative symptoms in schizophrenia. Consequently, a number of glycine modulators have been studied in clinical trials. In humans, the GlyT1 inhibitor ORG25935 reduced the ketamine-induced increases in measures of psychosis and perceptual alterations with an effect size of 0.71 and 0.98, respectively, but worsened some aspects of learning and delayed recall (D'Souza et al., 2012). Studies with the GlyT1 inhibitor GSK1018921 suggested that target engagements up to 80% were well tolerated (Ouellet et al., 2011). The Janssen GlyT1 inhibitor R213129 enhanced scopolamine-induced finger tapping impairment in healthy volunteers, while electroencephalography alpha power was increased and scopolamine-induced impairment of the Stroop test was partly reversed (Liem-Moolenaar et al., 2010). The Pfizer GlyT1 inhibitor PF03463275 was ineffective at the highest dose (NIH NCATS website http://www.ncats.nih.gov/research/reengineering/rescue-repurpose/therapeuticuses/directory.html).

In a meta-analysis with 800 subjects from 26 studies, glycine, D-serine, and sarcosine had effect sizes ranging from 0.40 in negative symptoms to 0.28 for cognitive and 0.26 for positive symptoms, whereas D-cycloserine did not improve any symptom domain. Interestingly, patients on risperidone or olanzapine, but not clozapine, improved (Tsai et al., 2004).

Glycine directly activates the glycineB site on the NMDA-R, but needs to be given in large quantities; D-serine is another endogenous activator of the NMDA-R on a different binding site and sarcosine was found to be a GlyT1 inhibitor (Wolkenberg and Sur, 2010). The absence of target engagement data in these clinical trials makes it difficult to interpret the clinical outcome.

Preclinical data on cognitive effects together with target engagement studies in non-human primates for two GlyT1 inhibitors strongly suggest an inverse U-shape dose-response (Eddins et al., 2014); in this study the highest doses consistently failed to improve cognition and bitopertin was found to be effective only at the lowest and medium doses, but not at the highest dose. An inverse U-shape dose-response is a difficult property for any clinical trial development; although such a dose-response is often observed in preclinical animal models, it is often difficult to relate this to actual human target engagement levels. Therefore, exploring the neurophysiology of such complex dose-responses in a humanized translational model is of crucial importance to drug development. In this report we will use an *in silico* quantitative systems pharmacology model (Geerts et al., 2013b) that integrates preclinical information with clinical neuropathology, imaging, and clinical data and that has been successful for cognitive enhancements in schizophrenia (Geerts et al., 2013a) and Alzheimer's disease (Roberts et al., 2012; Nicholas et al., 2013) and for motor side-effects of new antipsychotics (Geerts et al., 2012).

The remainder of the introduction will be devoted to the biological rationale for identifying the brain regions and neurophysiological processes that play a role in the clinical phenotype of negative symptoms. Unlike preclinical animal models, we will use predominantly imaging studies from patients and their relationship to clinical scales.

### Biological rationale for computer model of negative symptoms

#### Brain regions/neurophysiology involved in negative symptoms

The prefrontal cortex and ventral striatum are key brain regions involved in the processing of negative symptoms. From ASL-fMRI imaging studies to measure cerebral blood flow (CBF) in schizophrenic patients on antipsychotics medications (Pinkham et al., 2011), hypofrontality was most prominent in individuals with more severe negative symptoms. A large meta-analysis of 25 imaging studies (Goghari et al., 2010) suggests an inverse correlation between BOLD-fMRI activity of the ventromedial cortex and the degree of negative symptoms. Metabolic activity, measured by PET imaging, is reduced as negative symptoms increase in patients without antipsychotics (Wolkin et al., 1992) and physical anhedonia scale scores were negatively correlated with the hypoactive dorsomedial PFC metabolism (Park et al., 2009).

Another study suggests that activity of R. orbitofrontal cortex, but not anterior cingulate correlates with the self-reported Chapman Physical Anhedonia Scale (Harvey et al., 2010). As anhedonia together with avolition and apathy form the more “experiental” factor in negative symptoms, as opposed to flat affect that is more “expressive” (Horan et al., 2011); this suggests that lower activity of the R. orbitofrontal dysfunction might play a role in negative symptoms. Moreover, an inverse correlation of negative symptoms with R. anterior prefrontal cortex activity at rest (Mingoia et al., 2012) suggests that basal cortical activity is proportionally lower in patients with predominantly negative symptoms but the identity of the cortical region depends upon the task involved or the measurement condition.

This overview suggests that the cortical activity especially of the vmPFC and the right orbitofrontal cortex is lower in schizophrenia patients, and that increased activation might correspond to improved symptoms.

Imaging studies of ventral striatum pathology in schizophrenia (Menon et al., 2001; Harvey et al., 2010) suggest a profound and proportional dysfunction, with more negative symptoms associated with decreased activation level. In patients, lower ventral striatum activation in patients is proportional to the severity of negative symptoms, an effect that is independent of medication (whether medication-free, on typical or atypical antipsychotics) (Juckel et al., 2006a,b). In schizophrenia patients in psychotic remission (Sorg et al., 2013) basal activity of the ventral striatum is increased and this increase is correlated with improvements of negative symptoms such as emotional withdrawal and blunted affect.

#### Cellular localization of NMDA-NR2 subunits

The activity of the cortical region is driven by pyramidal cell firing in general and by glutamatergic action in particular. Therefore, NMDA-R is an interesting target for negative symptoms. However, because the cortical activity is defined by the balance of excitation over inhibition, it is of interest to take into account the differential localization of NMDA-R on pyramidal cells and interneurons in cortical circuits. mRNA localization studies of different NMDA-NR2 subunits in the rat and mice hippocampus, suggest that NR_2C/2D_ are localized on inhibitory interneurons while NR_2A_/NR_2B_ seem to be more concentrated on pyramidal cells (Monyer et al., 1994). Functional evidence was provided by elimination of NR_2C_ subunit having no effect on the strongly rectifying NMDA current in pyramidal cells of the prefrontal cortex (Zhang et al., 2012).

Reelin deficient heterozygous mice showed significantly enhanced MK-801-induced locomotor hyperactivity and startle, which was associated with significant up-regulation of NR_1_ subunits, but down-regulation of NR_2C_ subunits in the frontal cortex (van den Buuse et al., 2012), suggesting that loss of activity on inhibitory neurons through reduced NMDA-NR_2C_ synapses leads to a lower GABA tone, a functional disinhibition, and a higher locomotor activity. These and other preclinical data strongly suggests that while NR_2A_ and NR_2B_ are expressed on pyramidal excitatory cells, the NR_2C_ subunit is localized on inhibitory neurons.

#### Change of NMDA subunits with schizophrenia pathology

The NR2B subunit is upregulated during neurodevelopment of the brain and is likely to play a relatively larger role in schizophrenia, in line with the neurodevelopmental hypothesis of schizophrenia pathology. Indeed in postmortem dorso-lateral prefrontal cortex samples of schizophrenia patients vs. healthy control, increased phosphorylation of NR_2B_ at Y1336 is found (Funk et al., 2012), probably leading to a higher functional activity by reducing endocytosis (Jiang et al., 2011; Li et al., 2011). In patients with schizophrenia, a significant effect of GRIN2B (human NMDA receptor 2B subunit gene, NR_2B_) genotype on habituation (Hokyo et al., 2010) suggests a bigger role for NR_2B_ mediated processes.

Altered expression of mRNA for proteins involved in in microtubule-associated tracking complex of NR_2B_ such as KIF17, APBA1, CASK, mLin7A, and mLin7C in cortical layers III and IV of schizophrenia patients, which overlapped with NR_2B_ but not NR_2A_ transcripts suggests that NR_2B_-containing NMDA receptor transport could be selectively compromised in schizophrenia (Kristiansen et al., 2010a,b). In a subcellular endoplasmic reticulum (ER)-enriched fraction from postmortem brain, ER expression of NR_2B_ and PSD-95 was decreased in dorsolateral prefrontal cortex in schizophrenia. The data suggest that changes in NR_2B_ processing in schizophrenia involve increased ER exit of NR_2B_ containing NMDA receptors suggesting a higher membrane expression level (Kristiansen et al., 2010b).

Furthermore, a cross-sectional study of over 900 human brains from the publicly available BrainCloud website (http://braincloud.jhmi.edu/) suggests an increase in cortical mRNA for the NR_2B_ subunit during the adolescent period (10–20 years) that reverts for older brains. This suggests that during neurodevelopment the NR_2B_ subunit is upregulated in the human brain but its expression tends to decrease with age.

In summary these data suggest that the NMDA- NR_2B_ subunit is upregulated in schizophrenia patients.

### Glycine transporter physiology

In order to estimate the range of free glycine level that can be readily achieved in the living human brain, we need to consider the neurophysiology of the glycine transporter T1, found mostly on astrocytes but also on neuronal cells and is a co-transporter system driven by the electrogenic movement of 2 Na^+^ and 1 Cl^−^ over the cell membrane at a slow turnover rate of 10/s (Cherubino et al., 2010). Kinetics follow Michaelis-Menten dynamics with K_m_ in the range of 10–20 uM (Okamoto et al., 2009; Cherubino et al., 2010).

The astrocyte membrane potential is in the range of −75 mV (Ma et al., 2014) and does not share the same temporal dynamics as neuronal cells. The membrane can depolarize substantially in the case of ischemic and traumatic brain injury (Strong and Dardis, 2005), but we assume that the astrocyte membrane potential is close to the equilibirum value in schizophrenia.

## Methods

### Receptor competition model

Many antipsychotic drugs on the market have different affinities for multiple receptors, therefore calculating the receptor change for a given exposure level of the drug at each of these receptors is important, because they will affect the membrane potential of key neuronal circuits and their emergent properties.

The receptor model simulates the competition between endogenous neurotransmitter and up to four agents, (for instance two drugs with their metabolites or a drug and radioactive tracer) at postsynaptic receptors with full presynaptic autoreceptor coupling to neurotransmitter release based on the affinities of the drug for all receptors in the synaptic cleft (Spiros et al., 2010). This is performed using a set of ordinary differential equations that takes into account different neurotransmitter release patterns and modulated by presynaptic autoreceptors, including presynaptic facilitation and depression processes. The dopaminergic synapse is further calibrated (Spiros et al., 2010) using data on dopamine dynamics measured with fast cyclic-voltammetry in monkey slices (Cragg et al., 2000) and human cortical imaging data (Slifstein et al., 2008), while the serotonin synapse with 5-HT_1B_ as a presynaptic autoreceptor is calibrated using a combination of preclinical fast cyclic voltammetry constrained by human imaging data (Roberts et al., 2012).

The affinity parameters for each antipsychotic and neurotransmitter for human receptors were derived from the *in vitro* experiments performed at the Psychoactive Drug Screening Program (PDSP) and reported in the PDSP database (http://pdsp.med.unc.edu/indexR.html) with the advantage that the affinity values are derived under the same standardized assay conditions. For different values of target engagement (e.g., D_2_R occupancy), we then calculated the change of postsynaptic receptor activation for all the receptors involved in the computer model based on the affinities of the drug for different receptors.

### Cortical-striatal model for negative symptoms

**Figure 1:**
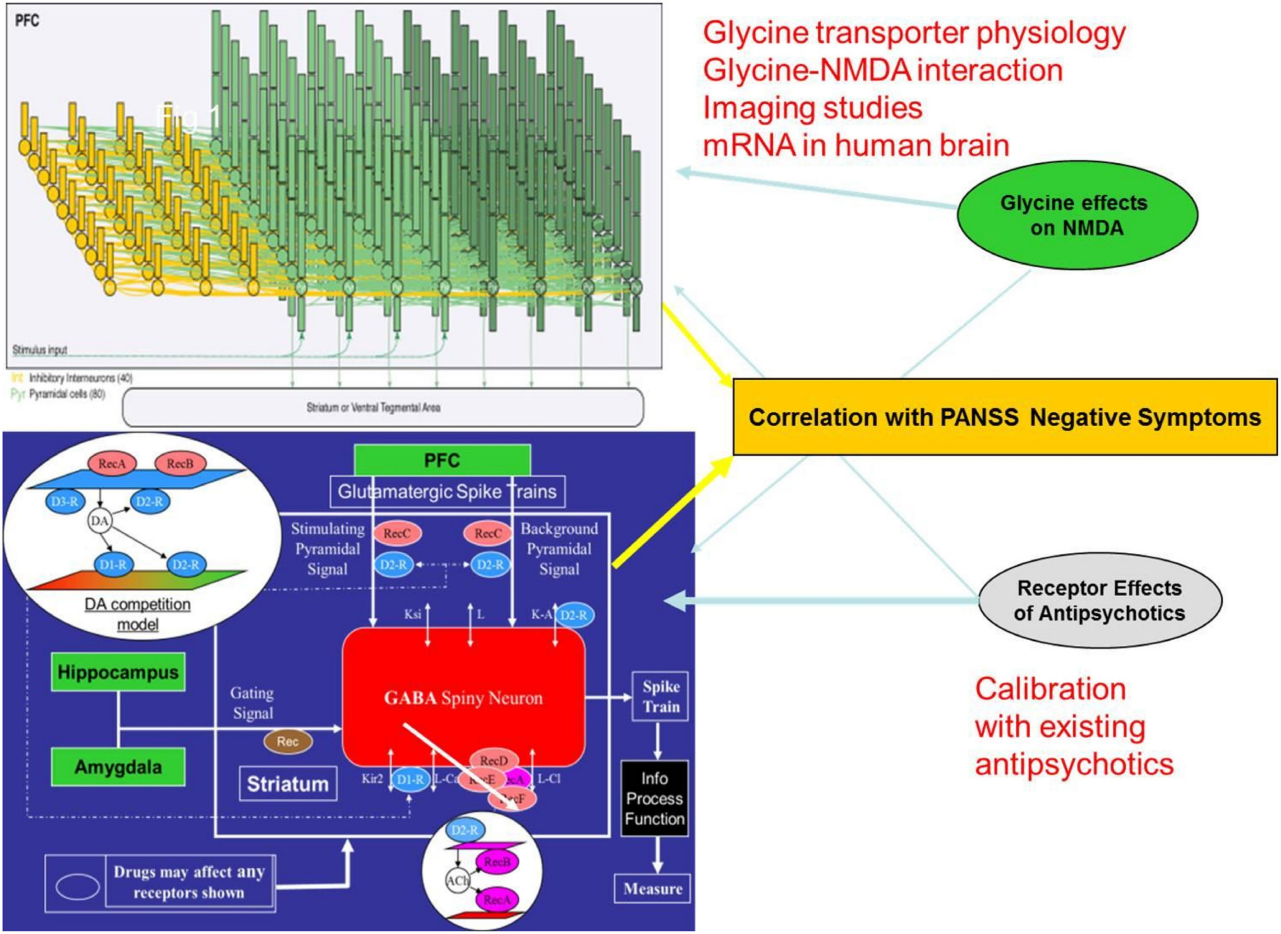
**Schematic description of the two-part quantitative systems pharmacology model for negative symptoms of schizophrenia**. The BOLD-fMRI signal of a neuronal cortical circuit (top) is calculated after implementing a representation of the schizophrenia pathology and the effect of glycine modulation. This is combined with the glycine BOLDfMRI effect through the afferent NMDA-dependent projections of the cortical input in a model of the ventral striatum medium spiny neuron (bottom). Combining the output of these two models results in a weighted readout that is a human imaging based proxy for the clinical phenotype of negative symptoms. While currently marketed antipsychotics exert their effects on PANSS negative mainly through the ventral striatum part, glycine modulation acts mostly through the cortical network. The simulation of historical trials with the appropriate drug-dose relationship of currently marketed antipsychotics in this model and comparison with the reported clinical changes on the PANSS negative subscale provides a calibration relation where the anticipated clinical effect of new therapeutic interventions can be predicted from the outcome in the computer model.

Based on the human imaging studies, we developed a dual cortical-striatal model for the neurobiology of negative symptoms (Figure 1). The cortical neuronal network consists of 20 excitatory neurons and 10 inhibitory interneurons and has been described before (Geerts et al., 2013a). This model has been calibrated from *in vivo* single-unit recordings in primates during a working memory task and reduces some of the problems associated with species difference in inhibitory tone. Synchronous firing of the target pyramidal cells is initiated by injecting a transient current at *t* = 2000 ms. The network then fires in a synchronized pattern before it gets degraded by the background noise and the interference of the distractor neurons. The simulated neural activity represents the right orbitofrontal cortex or the vmPFC.

Functional representations, driven by preclinical experiments on the coupling between receptor activation and changes in voltage-gated ion channel conductance, of the dopamine (D_1_, D_2_, D_3_, D_4_, DAT, COMT), serotonin (5HT_1_A, 5HT_1_B, 5HT_2_A, 5HT_2_C, 5HT_3_, 5HT_4_, 5HT_6_, SERT), norepinephrine (alpha_1_A, alpha_2_A, NET), cholinergic (M_1_ mAChR, M_2_ mAChR, α_7_ nAChR, α_4_β_2_ nAChR, and AChE), glutamate NMDA (different subunits NR2A-NR2B-NR2C), AMPA, mGluR2, mGluR5, GlyT1, GABA-A α_1_ and GABA-A α_2_, histamine H_3_ and PDE-10 targets are currently implemented in the model.

Although the intracellular pathways activated by receptor modulation are not modeled in full detail, we implement the effects as a transfer function on ion channel permeability or transporter functionality. For instance a change in dopamine D_1_R activation on cortical neurons is implemented by changing the slow K^+^ channel *I_ks_* conductance (Yang and Seamans, 1996) and the High-voltage activated (Hva) Ca^++^ -channel, based on preclinical electrophysiological measurements (Law-Tho et al., 1994).

Schizophrenia pathology in the cortical network is introduced as a reduction in glutamate tone (Coyle, 2006), decreased dopamine tone (Meyer-Lindenberg et al., 2002; Weinberger, 2007) in the cortex, impaired GABA physiology through a decrease in GAD67 activity resulting in lower GABA release (Gonzalez-Burgos et al., 2010) and increased background noise level (Winterer et al., 2000). Such a pathology when implemented in the computer model leads to a deterioration of a marker for cognitive outcome of about 1.5 standard deviations (Geerts et al., 2013a). In this case, rather than the length of time a certain firing pattern can be independently held, the cortical readouts of the model for negative symptoms of schizophrenia is the average firing rate and BOLD-fMRI.

The ventral striatum model has been described in detail as part of the quantitative systems pharmacology platform for schizophrenia (Geerts et al., 2012; Spiros et al., 2012). Briefly, the model calculates the excitability of the medium spiny neuron (MSN), the major GABA-ergic cell type in the nucleus accumbens, when driven by afferent cortical projections and gated by both hippocampal and amygdala projections. Changes in membrane potential are calculated using partial differential equations that are solved in NEURON (Hines and Carnevale, 1997). If C is the membrane capacitance, then the time course of the membrane potential V can be determined from the following equation:

(1) $$C\frac{\partial V}{\partial t}=I_{Ksi}+I_{KA}+.I_{Cl}+I_{Ca}+\ldots$$

where *I_X_* is the current associated with channel X.

We simulate three types of neuronal MSN cells: SP^+^ = D_1_R^+^ cells that project to the direct pathway; Enk^+^ = D_2_R^+^ cells that project to the indirect pathway; and a small number of D^+^_1_D^+^_2_ cells that project to both pathways. In the SP^+^ cells the D_1_R mostly affects the *K_ir2_* channel and increases the L-type Ca^++^ current (Hernandez-Lopez et al., 2000), while in Enk^+^ cells, D_2_R activation affects the A-type K^+^ current (Falk et al., 2006). In addition, D_2_R activity modulates the presynaptic Glu release on the afferent cortical fibers (O'Donnell and Grace, 1994; Bamford et al., 2004).

For instance, the inward rectifying potassium current, *Kir*2, is modified by the dopamine D_1_R activation u (Kuzhikandathil and Oxford, 2002; Falk et al., 2008) so that the total current, *I* = *u* · *I*_*Kir*2_. With a conductance, *g_K_*, and a reversal potential, *E_K_* = −90 mV, the current is given by *I*_*Kir*2_ = *g_K_* (*V* − *E_K_*) with a voltage dependent form

(2) $$g_{K}={\bar{g}}_{K}\frac{1}{1+\exp\left(-\frac{V-V_{h}^{K}}{V_{c}^{K}}\right)}$$

where *g*_*K*_ = 1.2 mS/cm^2^ is the maximum conductance, *V_h_* = −111 mV is the value of the membrane potential that causes half activation and *V_c_* = −11 mV describes the sensitivity of the change (Mermelstein et al., 1998; Gruber et al., 2003).

The amount of DA released in the striatal dopaminergic synapse is increased by 5-HT_2C_ receptor inhibition (Abdallah et al., 2009), while 5-HT_3_R antagonism decreases striatal DA (De Deurwaerdere et al., 1998; Porras et al., 2003). Cholinergic modulation affects the excitability of MSN through an effect on Cl- channel (Shen et al., 2005, 2007) through postsynaptic M_1_R mAChR. In addition, M_2_ mAChR located on corticostriatal terminals (Hersch et al., 1994) inhibit the glutamatergic input to MSNs (Malenka and Kocsis, 1988; Sugita et al., 1991; Calabresi et al., 1998; Hernandez-Echeagaray et al., 1998). Adrenergic alpha_1A_-R block decreases gating signal stimulation of the GABA spiny neuron (Braga et al., 2004; Aroniadou-Anderjaska et al., 2007). All these processes are implemented using the appropriate differential equations with a linear relationship between the increase of DA and normalized activation level.

### Implementation of the bold-fMRI readout

In order to calculate a measure of the BOLD-fMRI outcome from the computer model, we implemented a series of biophysical relations between excitatory and inhibitory neuronal activity as determined by experimental studies (Sotero and Trujillo-Barreto, 2007, 2008). The relevant equations are implemented describing the relationships between excitatory and inhibitory neuronal activity, glucose consumed, oxygen consumed, and CBF changes to obtain a measure of the BOLD signal with the Balloon model (Buxton et al., 2004; Buxton, 2012) with the parameters provided from a review study (Sotero and Trujillo-Barreto, 2008).

With *v*(*t*) the normalized cerebral blood volume, *f*(*t*) the normalized CBF and *q*(*t*) the doxyhemoglobin content, the BOLDfMRI signal *y*(*t*) is described by

(3) $$y\left(t\right)=V0(a_{1}\left(1-q\right)-a_{2}(1-v)$$

(4) $$\text{With}\frac{dv\left(t\right)}{dt}=\frac{1}{t_{0}}\left(f\left(t\right)-f_{out}\left(v,t\right)\right)$$

(5) $$\frac{dq\left(t\right)}{dt}\;=\frac{1}{t_{0}}(m\left(t\right)-f_{out}\left(v,t\right)\frac{q\left(t\right)}{v\left(t\right)})$$

(6) $$fout\left(v,t\right)=v\exp\left(\frac{1}{a}\right)+\tau dv(t)/dt$$

Furthermore, with *m*_*e*_(*t*) and *m*_*i*_(*t*) the metabolic rate of oxygen consumption from excitatory and inhibitory cells, respectively

(7) $$m\left(t\right)=(\gamma m_{e}\left(t\right)+m_{i}\left(t\right))/(\gamma +1);$$

(8) $$m_{i}\left(t\right)\;=g_{i}(t)\text{ and }m_{e}\left(t\right)=g_{e}(t)(2-x\left(t\right))/(2-xo)$$

(9) $$\text{And }g\left(t\right)=(2\gamma g_{e}\left(t\right)+\left(2-xo\right)g_{i}\left(t\right))/(2\gamma +2-xo)$$

With *u*_*e*_(*t*) and *u*_*i*_(*t*) the excitatory and inhibitory neuronal activity, both *g*_*e*_(*t*) and *g*_*i*_(*t*), the glucose level normalized to baseline consumption, are further defined by

(10) $$\begin{array}{l} \;\frac{dg_{e}\left(t\right)}{dt}=s_{e}\left(t\right)\\ \text{and }\frac{dg_{i}\left(t\right)}{dt}\;=s_{i}(t)\text{ with} \end{array}$$

(11) $$\frac{ds_{e}\left(t\right)}{dt}\;=\frac{a_{e}\left(u_{e}\left(t-\delta_{e}\right)-1\right)}{\tau_{e}}-\frac{2s_{e}\left(t\right)}{\tau_{e}}-\frac{g_{e}\left(t\right)-1}{\tau_{e}*\tau_{e}}$$

With an identical equation for *s*_*i*_(*t*) with all indexes referring to inhibitory interneuron activity *u*_*i*_(*t*).

The CBF f(t) is defined by $\frac{df\left(t\right)}{dt}=s(t)$.

Where $\frac{ds\left(t\right)}{dt}=\varepsilon \left(e_{e}\left(t-\delta_{f}\right)-1\right)-\frac{s\left(t\right)}{\tau_{s}}-(f\left(t\right)-1/\tau_{f}$.

Values for the different constants are given in Table 3 of Sotero and Trujillo-Barreto (2008). For instance, *a_e_* is the efficacy of glucose consumption response to excitation (1.2); *c* the steepness of the sigmoid function (2.5) and *d* the position of the threshold for the sigmoid function (1.6), τ the time constant that controls how fast Cerebral Blood volume adjusts to changes in CBF (10 s), *a*_1_ weight for deoxyhemoglobin change (3.4) and a_2_ weight for blood volume change (Rosenbaum et al., 2012).

### Implementation of the glycine neurophysiology in the model

The ratio of NR_2A_/NR_2B_ subunit on pyramidal excitatory synapses vs. NR_2C_/NR_2D_ subunit on inhibitory cells is an important driver of glycine modulation. Glycine interacts differently with different NMDA-NR_2_ subtypes even if the binding site is on the NR_1_ subunit. The potentiation of NMDA current by glycine has been measured experimentally and can be described by a Hill equation. The NMDA-R conductance g can be described as follows.

(12) $$g=g_{\max}\frac{{\left[Gly\right]}^{n}}{{\left[Gly\right]}^{n}+{\left[EC_{50}\right]}^{n}}$$

With *g*_max_ a maximal conductance value, [*Gly*] the concentration of extracellular glycine and with numerical values for EC_50_ and Hill slope determined from a number of experimental studies (Kutsuwada et al., 1992; Laurie and Seeburg, 1994; Matsui et al., 1995; Woodward et al., 1995; Chen et al., 2008).

The EC_50_ (concentration at which effect is 50% of maximum) and Hill slopes (*n*) for different experimental conditions are given in Table 1. It will be clear that with different values for the Hill equation and EC_50_ we get rich dynamics in terms of the ratio of the NMDA currents on pyramidal-pyramidal synapses (mostly NR_2A_-NR_2B_) vs. the NMDA currents on pyramidal-inhibitory synapses driven by the NR_2C_-NR_2D_ subunits (see Figure **3** for example).

**Table 1:** **Experimentally determined values for EC_50_ and Hill slopes for glycine-glycine site interaction on the NMDA-NR_2_ subunit from different experimental conditions**.

**Parameter**	**NR_2A_**	**NR_2B_**	**NR_2C_**	**NR_2D_**	**References**
EC_50_ (uM)	0.97	0.84	0.75	0.56	Matsui et al., 1995
Hill slope	1.5	2	2	1	
EC_50_ (uM)	2.1	0.3	0.2		Kutsuwada et al., 1992
Hill slope	1.5	1.5	1.5		
EC_50_ (uM)	0.84	0.19	0.15	0.096	Woodward et al., 1995
Hill slope	1.5	2	1.5	1	
EC_50_ (uM)	3.7	2.1	0.36	2.3	Laurie and Seeburg, 1994
Hill slope	1.	1.5	1.5	1.5	
EC_50_ (uM)	1.31	0.72	0.34	0.13	Chen et al., 2008
Hill slope	1.66	1.84	1.81	1.32	
Average EC_50_ (uM)	1.78	0.83	0.36	0.77	
Average Hill slope	1.43	1.77	1.66	1.21	

*EC_50_ values are definitely higher and there is a trend for higher Hill slope for excitatory-excitatory NR_2A/B_ subunits (especially when considering the NR_2B_ subunits) over the excitatory-inhibitory NR_2C/D_ subunits*.

### Calibration of the model with clinical data on negative symptoms

The model is subsequently calibrated using historical clinical trials. Historical clinical data in schizophrenia patients were collected by querying PubMed with the keywords “drug X” and “schizophrenia” and “trial” in the period since 1986. Restricting the data to clinical double-blind placebo-controlled studies on drug monotherapy using stable schizophrenia patients for a short duration (4–12 weeks), resulted in 91 papers and 71 drug-dose combinations. For each drug dose combination, we calculated the change in postsynaptic receptor activation using the receptor competition model using the appropriate affinities of the neurotransmitter, the drug and its metabolite.

We assume a linear normalized relationship between receptor activation and biological effect on physiological responses such as $X_{Y}^{eff}\;=\frac{X_{Y}^{A}-X_{Y}^{C}}{X_{Y}^{C}}$; (Equation 13) where *X^A^_Y_* and *X^C^_Y_* are the actual activation levels of receptor X subtype Y (for instance D_1_) after treatment (A) and the untreated (placebo) control levels (C).

Such short clinical studies are common in the clinical testing of antipsychotics and motor side effects can arise very early with treatment. For each study, the average outcome of a patient group on the reported clinical trial was entered into a database. In the case of multiply reported results for the same drug-dose, the weighted average outcome based on number of patients was calculated. The list of clinical studies can be requested from the corresponding author.

## Results

### Calibration of the model for negative symptoms outcome

Extraction of the relevant information from the clinical database results in 34 drug-dose combinations of short-term clinical trials (4–12 weeks) with outcomes on the PANSS negative subscale. Each of these drug-dose combinations was then first simulated with the appropriate PET tracer displacement (with ^11^C-raclopride) studies in the dopamine receptor competition model to yield the functional intrasynaptic drug concentration that corresponded to the observed tracer displacement. These functional drug doses were then applied to the receptor competition model for all postsynaptic receptors (other dopamine receptors, in addition to serotonergic, cholinergic, and norepinephrine receptors) in the computer model using the appropriate pharmacological interaction between that particular drug and the human receptor subtype. With the resulting changes in postsynaptic receptor activation for all synapses in the negative symptoms QSP computer model, *in silico* computer model results were obtained for each drug-dose combination, in particular changes in BOLDfMRI readout of the cortical part of the computer model and activity in the ventral striatum computer model. A weighted combination of these two parameters was then defined as a proxy for negative symptoms.

**Figure 2:**
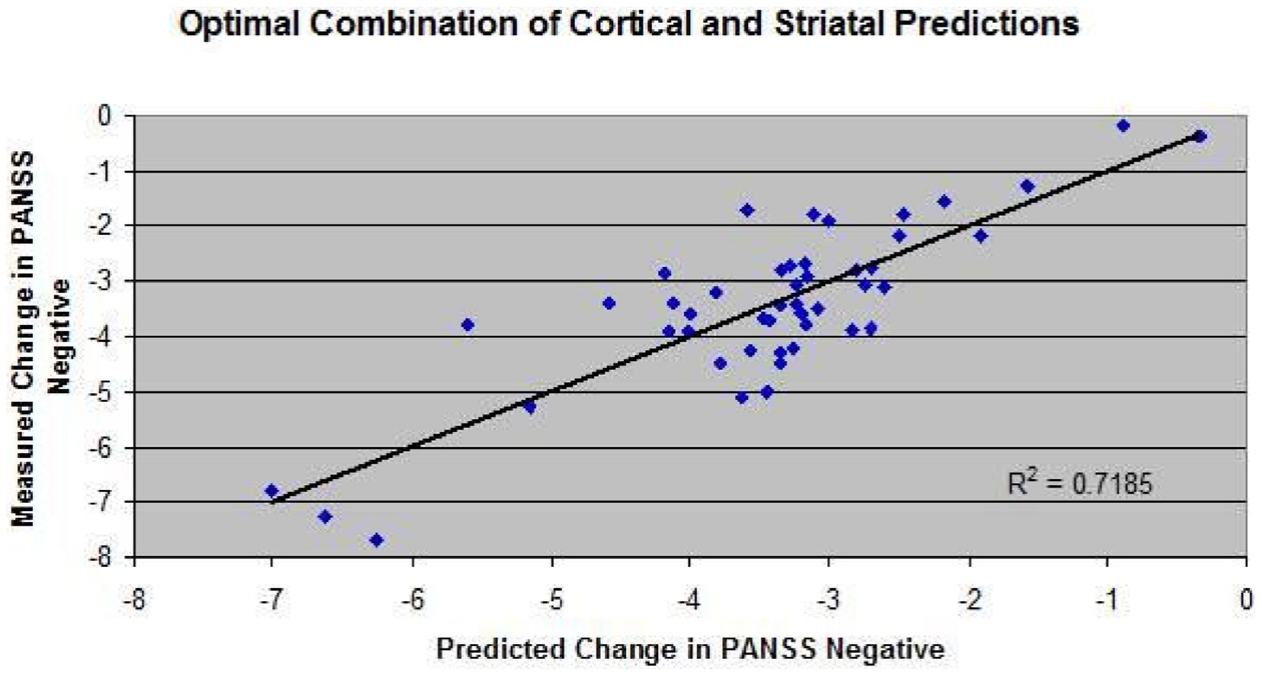
**Correlation between model outcome and changes on PANSS negative clinical scale for 34 drug-dose combinations derived from the literature since 1988 on pivotal Phase III or head-to-head comparison trials**. For each drug dose-combination, the appropriate target engagement (derived from PET radiotracer displacement studies) and pharmacology against human receptors was used to calculate the impact on changes in all postsynaptic receptors in the model, leading to changes in BOLDfMRI readout of the cortical part of the computer model and activity in the ventral striatum computer model. A weighted sum of these two parameters (40% cortical input and 60% ventral input) was defined as a proxy for negative symptoms. In addition, a few biological coupling parameters in the ventral striatum model were adjusted to achieve a robust correlation between model results and clinically reported outcomes.

Those results were then compared to the corresponding actual clinical readouts on PANSS Negative. Figure 2 shows the correlation between the model outcome of these drug-dose combinations and the actual reported changes in PANSS Negative. A few biological coupling parameters in the ventral striatum model were adjusted to achieve a robust correlation. The observed correlation suggests that the model captures a substantial part of the variance.

### Dose-response of glycine modulation

**Figure 3:**
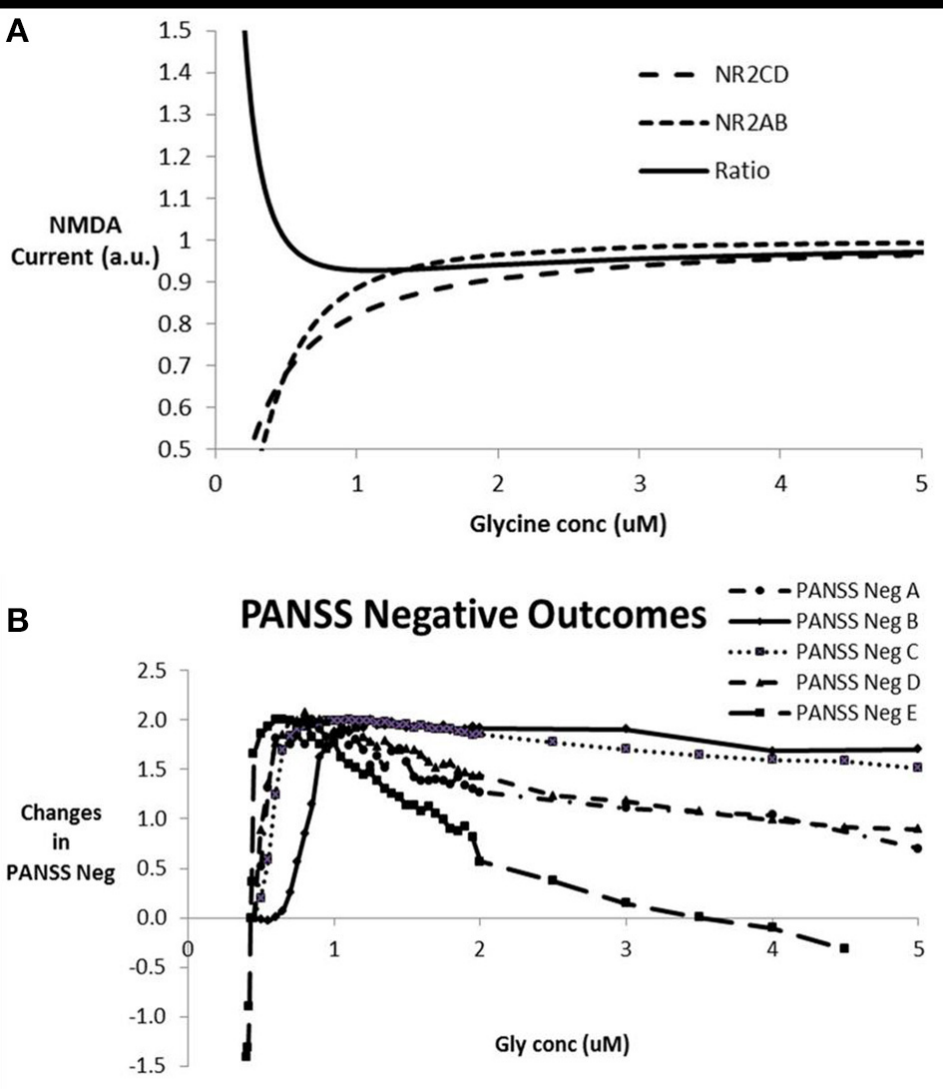
**(A)** Relative currents through NMDA- NR_2C/D_ subunits in pyramidal-inhibitory glutamatergic synapses (e-i) over NMDA currents and NMDA- NR_2A/B_ subunits in pyramidal-pyramidal glutamatergic synapses (e-e) and the ratio of NR_2C/D_ over NR_2A/B_ currents as a function of glycine concentration. This particular graph corresponds to a value of 0.35 and 0.25 uM for EC_50_ of NR_2A/B_ and NR_2C/D_ respectively and a Hill coefficient of 1.84 and 1.1 for NR_2A/B_ and NR_2C/D_ respectively. This particular case shows a minimum of 0.93 on the ratio of NR_2*C*/*D*_ over NR_2*A*/*B*_ at a glycine concentration of 1.1 uM. For certain combinations of the EC_50_ and Hill coefficients of the interaction between glycine and its binding site on NMDA receptors, the ratio shows a U-shape dose-response. For glycine concentrations where the e-i over e-e ratio drops below 1 (in this case between 0.5 and 30 uM), this leads to a relative dominance of the excitatory tone over the inhibitory tone and a greater firing and BOLD-fMRI outcome. **(B)** Dose-response of the model outcome for negative symptoms as a function of glycine concentration for different combinations of parameters for glycine-NR_2C/D_ vs. glycine-NR_2A/B_ interactions after calibration with clinical data. The parameter settings are EC_50_ for NR_2C/D_, Hill coefficient for NR_2C/D_, EC_50_ for NR_2A/B_, Hill coefficient for NR_2A/B_, respectively so that the conditions shown are A (0.2 uM, 0.7, 0.3 uM, 1.7); B (0.2 uM, 1, 0.22 uM, 1.5); C (0.21 uM, 1, 0.25 uM, 2); D (0.2 uM, 1, 0.35 uM, 2); and E (0.20 uM, 1.46, 0.35 uM, 1.78). In a clinical trial, glycine concentration can be altered using either a glycine transporter inhibitor or substitution with glycine. Note the inverse U-shape dose-response that parallels the ratio of current through the NMDA-NR_2_ subunits located at the excitatory-excitatory synapses over the current through the NMDA-NR_2_ subunits located at the excitatory-inhibitory synapses. Maximal effect is in the two-point range but is only obtainable if the typical glycine concentration is around 1 uM. With higher glycine concentration, in many cases the effect decreases, with a worsening of negative symptoms in some cases for glycine concentrations greater than 5 uM.

In order to simulate the effect of modulating glycine concentration on the network outcome, we proceeded by calculating the Hill equations for the interaction between glycine and the NR_2_ subunit on the excitatory-excitatory (e-e) glutamatergic synapses with NMDA subunits (NR_2A_-NR_2B_) and the excitatory-inhibitory (e-i) glutamatergic synapses with NMDA subunits NR_2C_-NR_2D_. Figure 3A shows the currents through the two synapse types and their ratios as a function of glycine concentration. Due to the complex non-linear effects, for a number of EC_50_ and Hill coefficient parameter settings, the ratio of inhibitory to excitatory NMDA response decreases, reaches a minimum and then increases again, allowing for a complex non-monotonic dose-response. The range of EC_50_ and Hill coefficient parameters for which this can be observed is examined in the following section.

When entering these changes for the effect of glycine on e-e and e-i glutamatergic synapses as corresponding changes in NMDA maximum conductances on the respective synapses, the impact on the output of the network can be calculated. Figure 3B shows the anticipated clinical outcome on the PANSS negative scale based on the calibration (Figure 2) for a number of combinations of the interaction parameters between glycine and its binding site on the NMDA receptor. The clinical benefit follows an inverse U-shape dose-response relationship with the free glycine concentration provided that the typical glycine concentrations are less than 0.5 uM, very similar to the dose-response of the ratio of the currents, NR_2C_/_2D_ to NR_2A_/_2B_ NMDA subtypes. In fact using a number of different values for the EC_50_ and Hill coefficient parameters, we can confirm the correspondence between these two dose-responses (data not shown).

Although the dose-responses in general are similar for the different glycine interaction parameters, the glycine concentration for which there is a clinical benefit varies substantially. As no experimental data are available for the interaction of glycine with the glycine binding site in the living human brain, in principle accordance between the model outcome and the clinical results will help to narrow down the range of likely parameters. In the following section we will address these issues in more detail.

### Sensitivity of the model to biological coupling and physiology parameters

We then studied the sensitivity of the model outcome as a function of small changes on all possible parameters, including the changes that reflect the implementation of the schizophrenia pathology. This could be envisaged as the intrinsic variability in a large set of patients possibly driven by the appearance of genotypic changes in neurophysiological and neuropathological pathways. For this we allowed the parameter to change by a certain fraction around their calibrated value. A general effect size is calculated by dividing the difference between the maximum and minimum relative effects by the fractional range in parameter settings. From the outcomes reported in Table 2, it is clear that the changes in pathology implementation at the level of the cortical network lead to the largest changes in the results. The effect of the parameters on the Hill equations for the interaction between glycine and its binding site on the NMDA-R will be explored in detail in the following section.

**Table 2:** **Sensitivity of the model outcome for different parameters and coupling constants that are changed between 20 and 50% in both directions for the glycine dose-response**.

	**Sensitivity range (%)**	**Minimal effect (%)**	**Maximal effect (%)**	**Effect size**
**INTERACTION GLYCINE**				
EC50 on inhibitory neurons	−50 to +50	−18.6	20.7	0.393
Hill slope on inhibitory neurons	+50 to −50	−22.1	13.4	0.355
EC50 on excitatory neurons	+50 to −50	−14.1	38.9	0.530
Hill slope on excitatory neurons	−50 to +50	−18.4	14.3	0.327
**CORTICAL SZ PATHOLOGY**				
NMDA reduction	+20 to −20	−37.7	18.2	1.398
GABA reduction	−10 to +10	−32.4	20.8	1.620
DA deficit	−41 to + 41	−7.9	2.8	0.130
Noise increase	+24 to −24	−13.9	18.5	0.675
**STRIATAL PHYSIOLOGY**				
DA receptors	+20 to −20	−9.3	10.1	0.485
Muscarinic receptors	−20 to +20	−3.5	15.6	0.478

*Maximal and minimal effect on the dose-response are derived. The general effect size is calculated as the difference between maximal and minimal outcome divided by the input range. The data suggest that GABA and NMDA reduction have a big impact on the outcome, likely because they are driving the baseline excitation-inhibition balance upon which glycine modulation will act*.

### Sensitivity analysis on glycine hill equation

A crucial set of parameters is the relative values of the EC_50_ and Hill coefficients for the glycine-NMDA current effect through the different NR2 subunits. As shown in the previous section, the inverse U-shape dose-response in the cortical network outcome corresponding to a clinical benefit on PANSS negative is associated with a U-shape dose-response of the ratio of inhibitory over excitatory effect (compare Figure 3A with Figure 3B). Note that the glycine concentration for maximal effect on the network corresponds to the glycine concentration of the minimum in the ratio of NR_2C/D_ over NR_2A/B_.

Because the experimentally determined values reported in Table 1 for the interaction of glycine with the human NMDA NR_2_ subunits were performed in an artificial *in vitro* system and could be quite different from the actual human *in vivo* situation, we systematically studied the effect of changing the values for EC_50_ on the network outcome.

**Figure 4:**
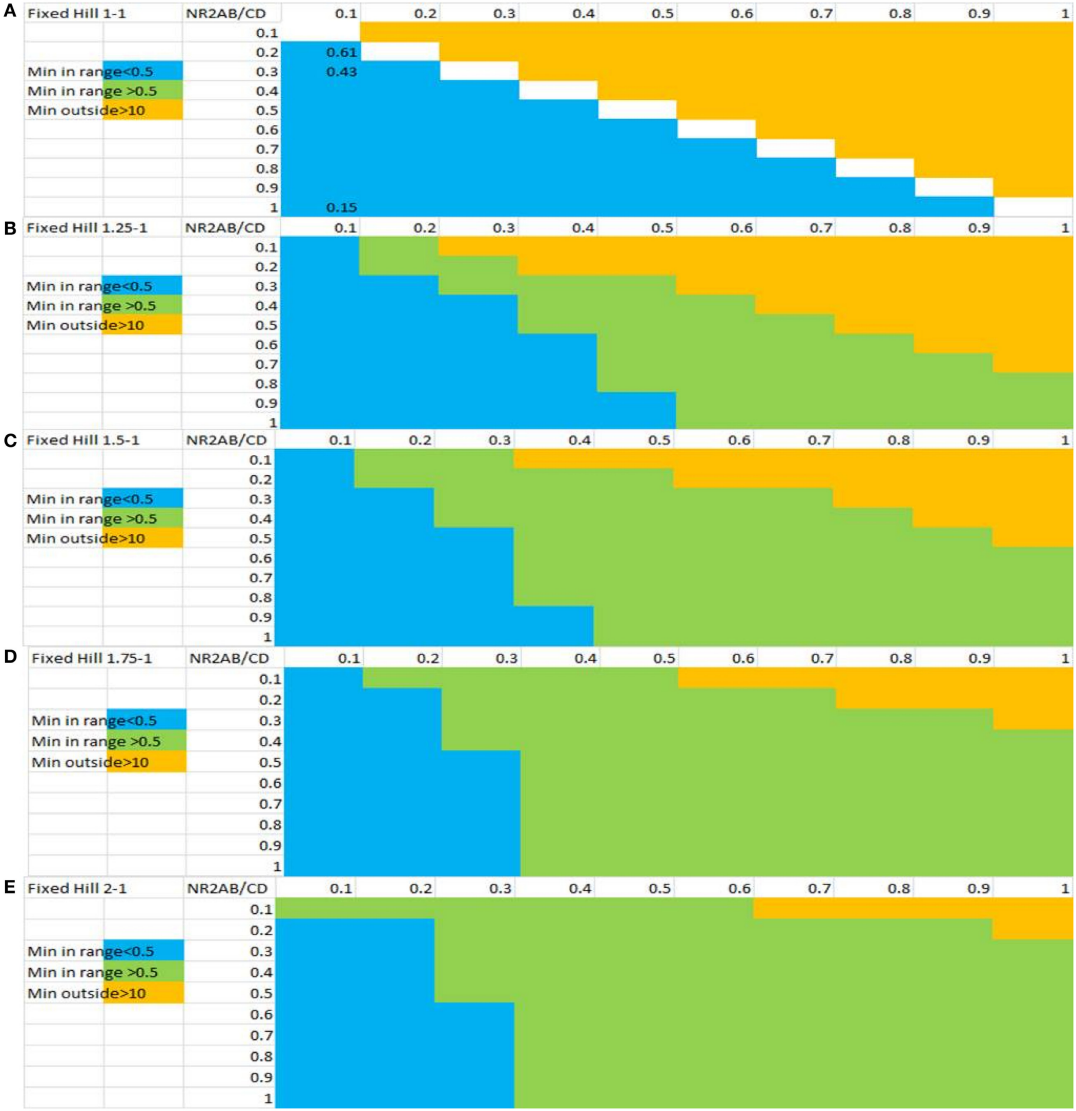
**Sensitivity analysis of the inverse U-shape dose-response for glycine-dependent outcome of the computer model as a function of the Hill slope and the EC_50_ values for both classes of NMDA-NR_2_ subunits**. The x-axis represents EC_50_ values for the e-i NR_2C/D_ subunits and the y-axis represents the EC_50_ values for the NR_2A/B_ subunits. Shown are ratios of different Hill coefficients on NR_2A/B_ vs. NR_2C/D_ subunits: **(A)** 1:1, **(B)** 1.25:1, **(C)** 1.5:1, **(D)** 1.75:1, and **(E)** 2:1. Combinations of values in green color are associated with a U-shape dose-response for NMDA effects of e-i over e-e with a minimum between 0.5 and 10 uM of glycine concentration and therefore a beneficial effect on the network outcome. Combinations in yellow are associated with values resulting in monotonic decrease of the ratio and combinations in blue are associated with a non-biological minimum much smaller than 0.5 uM. The results indicate that a beneficial inverse U-shape dose-response on negative symptoms is associated with large differences between the Hill coefficients for NR_2A/2B_ vs. NR_2C/2D_ subunits. In addition, in most cases, a beneficial inverse U-shape dose-response is associated with greater EC50 values for NR_2A/2B_ than for NR_2C/2D_.

Figure 4 shows the sensitivity analysis when probing different parameter ranges for EC_50_ and Hill coefficients of the interaction between glycine and the NMDA-NR2 subunits. It is clear that higher Hill coefficients for the NR_2A_/_B_ subunits compared to the NR_2C_/_D_ subunits is necessary for a beneficial effect on the network outcome with the range increasing with larger differences between the Hill coefficients for NR_2A_/_2B_ vs. NR_2C_/_2D_ subunits.

### Physiological range of glycine concentration

The free glycine concentration in the human brain is regulated by a 2Na^+^-Cl^−^-Gly co-transporter system and its value is constrained by the Nernst-Goldman equation. Therefore, the functional free glycine concentrations in steady-state equilibrium conditions is dependent upon the range of concentrations for intracellular and extracellular Na^+^ and Cl^−^. Assuming the glycine transporter is located on astrocytes, all calculations are done for astrocyte membrane potential and intracellular ion concentrations. Astrocyte membrane potential, while not changing on the same time scale as neuronal membrane potential is supposed to be in the −50 to −70 mV range for steady state conditions.

**Figure 5:**
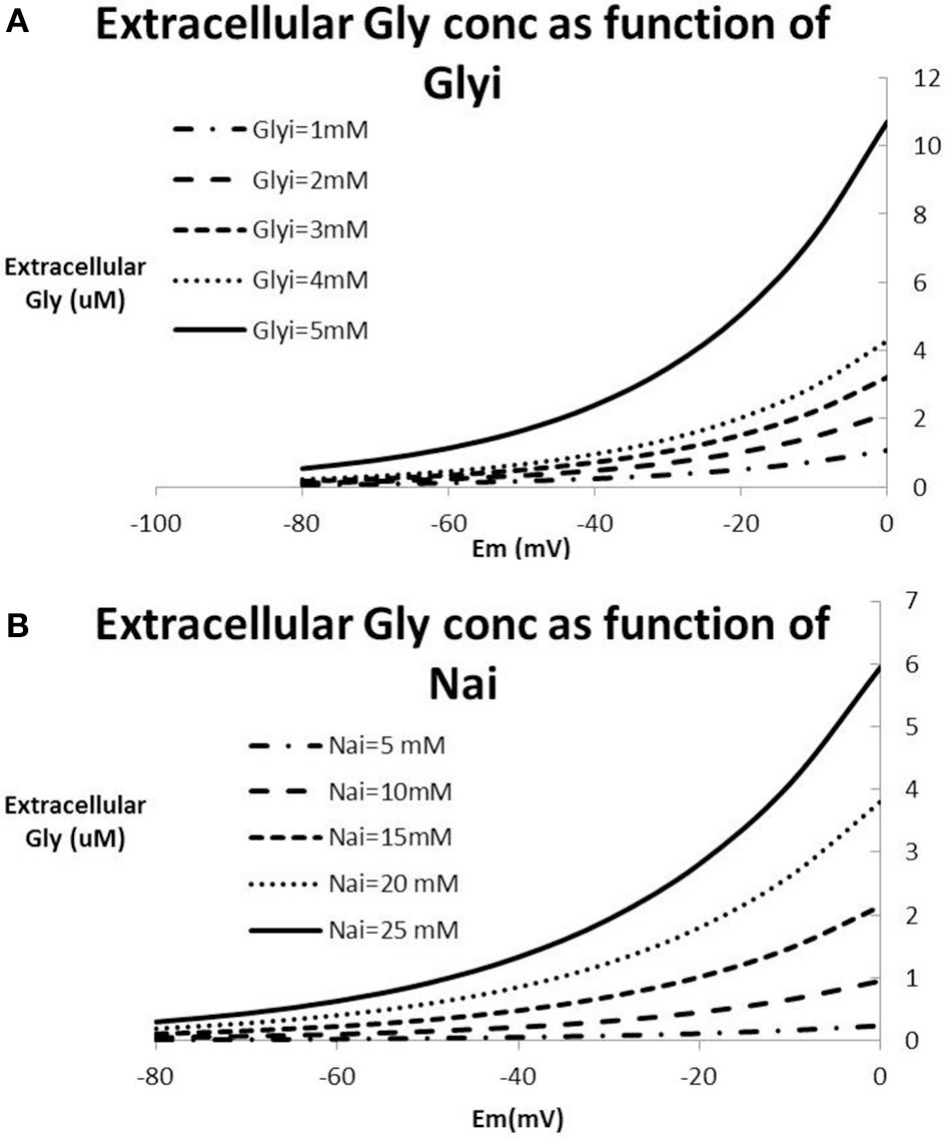
**Relationship between Na^+^-concentration, Cl^−^-concentration and glycine concentration based on the Nernst-Goldman equation that constrains the 2Na^+^-Cl^−^-Gly co-transporter system. (A)** Extracellular glycine concentration as a function of membrane potential for different values of intracellular Na^+^ keeping Gly_i_ fixed at 2 mM; **(B)** extracellular glycine concentration as a function of membrane potential for different values of intracellular glycine keeping Na^+^_i_ fixed at 15mM. All figures are derived for constant values of intracellular Cl^−^ of 12 mM and extracellular Cl^−^ concentration of 120 mM. With values of Na^+^_i_ in the range of 10–20 mM and membrane potential between −70 and −40 mV, this analysis suggests that extracellular glycine concentrations are limited to a range between 0.5 and 2–3 uM.

With the exception of extreme pathological situations such as in stroke or neurotrauma, Na^+^ and Cl^−^ ion concentrations in the human brain are tightly regulated. Figure 5 shows a number of solutions to the Nernst-Goldman equation for different ranges of intracellular Na^+^ and intracellular glycine concentrations. All figures are derived for constant values of intracellular Cl^−^ of 6 mM and extracellular Cl^−^ concentration of 120 mM. It is clear that in the absence of extreme pathology, the range of free extracellular glycine is limited and is unlikely to exceed 10 uM.

### Internalization of NMDA-R at high glycine concentrations

High glycine exposure in principle could overstimulate the NMDA-R and lead to epileptic seizures. In preclinical slice work, NMDA-R internalization has been observed at very high glycine concentrations, typically with an EC50 value in the range of 40 uM (Nong et al., 2003). While the Nernst-Goldman equations that regulate the free glycine concentration as a function of free Na and Cl strongly suggest that glycine concentrations beyond 5 uM are highly unlikely (see Figure 5), we nevertheless simulated the effect of NMDA-NR_2_ subunit internalization on the computer model outcome.

**Figure 6:**
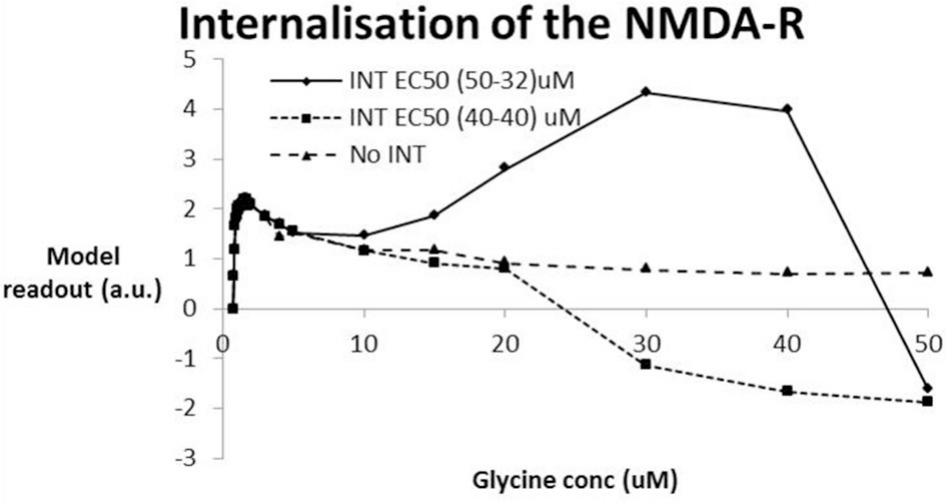
**Effect of NMDA-R internalization under the hypothesis that high levels of glycine are possible and using the experimental data from (Nong et al., 2003)**. Two conditions of internalization are simulated: INT-1–EC_50_ (40-40) assumes the same EC_50_ for internalization of NR_2A/B_ subunits as for NR_2C/D_ subunits (40 uM); INT-2–EC_50_ (50-32) assumes the EC_50_ for internalization of the NR_2C/D_ is lower (32 uM) than for the NR_2A/B_ subunit (50 uM) in accordance with the relative difference in EC_50_ for glycine to the glycine binding site. INT-1 is similar to the situation without internalization reaching a peak for glycine levels in the low uM until the network starts to collapse around a value of 20 uM. With INT-2, the model outcome reaches a similar early peak for glycine levels in the low uM that drops off before it begins to improve (at 10 uM) before collapsing at a glycine level beyond 40 uM.

We simulated two conditions of internalization (Figure 6). The first condition assumes the same EC_50_ value (40 uM) for both NR_2A_/_B_ subunits as for NR2C/D subunits. As expected, for very low levels of glycine where the internalization process has a very limited effect, the dose-response shows a peak for glycine levels in the low uM range. The results further suggest that internalization of the NMDA-R assuming the same EC_50_ values for the two types of NR_2_ subunits leads to a substantial decrease (corresponding to a collapse of network activity at glycine levels beyond 20 uM). The second assumption assumes that the internalization process would have the same affinity as glycine itself, i.e., an EC_50_ of 32 uM for the NR_2A_/_B_ subunits and an EC_50_ of 50 uM for the NR_2C_/_D_ subunits. In that case, the simulations suggest that the model outcome first improves beyond the no internalization case before collapsing at glycine levels beyond 40 uM.

## Discussion

This report describes a quantitative systems pharmacology computer model based on physiologically realistic interactions in models of a cortical network and the ventral striatum. The major result of this simulation is the prediction of an inverse U-shape dose-response with glycine that is a consequence of the shifting balance between excitation and inhibition in the cortical network, secondary to an interesting difference in pharmacological properties of glycine for the different NMDA subunits regulating excitatory and inhibitory tone.

The sensitivity analysis suggests that there are a substantial number of parameter combinations that result in such an inverse U-shape dose-response with glycine. The exact values for the interaction in the human brain is unknown and probably is different for each subject, but in general an inverse U-shape dose-response can be achieved when both the EC_50_ and the Hill coefficient for the glycine effect on the NR_2C/D_ subunit is lower than for the NR_2A/B_ subunit. It is of interest to note from different experimental data that on average the interaction of glycine with the NMDA-NR_2B_ subunit indeed suggests a higher value for the Hill coefficient. As noted in the Biological Introduction, the neurodevelopmental trajectory of schizophrenia tends to delay the appearance of the mature NMDA-NR_2A_ subunit, so that there is a relatively higher contribution of the NMDA-NR_2B_ subunit to the excitatory tone in schizophrenics. This allows the interaction of glycine with the e-e NMDA receptor to have a higher Hill slope in combination with a lower EC_50_ concentration, promoting an inverse U-shape dose-response.

The clinical Phase II data with the glycine inhibitor bitopertin suggest indeed that the clinical outcome follows such as dose-response. Some studies with D-serine, D-sarcosine or glycine have often reported mixed effects (Singh and Singh, 2011), with some but not all showing a clinical benefit and the interpretation is hampered by the lack of data on proper target engagement. It is conceivable that this could be a consequence of the non-linear dose-response with patients on different points of the dose-response. It is also of interest to note that negative symptoms seem to be most improved when glycine or D-serine levels are increased (Singh and Singh, 2011). The QSP platform when calibrated suggests a rather limited effect of glycine modulation on clinical changes in PANSS negative in the range of 2–2.5 points. Note that the patient population used for calibration was not selected for extremely high negative symptomatology, resulting in a baseline PANSS negative between 18 and 24. Although this observed change is in line with published data on glutamatergic modulation (Singh and Singh, 2011), it suggests that such a limited effect might be difficult to be detected in clinical situations.

These results also lead to the important observation that because of the different contribution of the NMDA-NR2 subunits, the interaction between glycine and the NMDA receptors likely is different in healthy volunteers as compared with schizophrenia patients. Such a difference is extremely difficult to achieve in preclinical animal models. Therefore, great care needs to be taken to extrapolate positive or negative findings from a Phase I proof-of-concept study in healthy volunteers to actual schizophrenia patients.

Similarly, rodent models often lack the right mix of NMDA receptor subtypes to simulate very well the actual pathology mediated and often do not show the inverse U-shape dose-response (Alberati et al., 2011). Not realizing this could substantially hamper the clinical development and often can lead to failed clinical trials. In addition, clinical trials have been performed as augmentation strategy, i.e., the glycine modulator intervention is given to patients on stable antipsychotic medication. Such comedications can have a direct effect through affecting the metabolism of the active compound which is dependent upon the genotype of the specific Cytochrome P450 enzyme but can also be modified by other comedications such as nicotine (Tsuda et al., 2014). The comedications can also have an indirect effect on the dose-response of glycine level modulation through non-linear interactions on the excitation-inhibition balance that affect the emergent properties such as the BOLD-fMRI signal. This paper does not address the issue of comedication, but we are planning to perform such an in-depth analysis in a follow-up paper.

### Limitations of the model

Firstly, different reports suggest that D-serine plays a more important role as co-agonist on the NMDA-R in the cortex (Fossat et al., 2012) while other studies suggest a role for both D-serine and glycine in regulating neuronal morphology in rodent somatosensory cortex (Balu et al., 2012). To a certain extent, the interaction of D-serine with the co-agonist site on the NMDA-R is quite similar to glycine's interaction (Chen et al., 2008), but the level of free D-serine is regulated by serine racemase localized in neurons (Balu et al., 2014) and by a Na^+^-independent alanine-cysteine-serine transporter system (Maucler et al., 2013). This suggests that most of the conclusions for glycine, with the exception of the limited range of glycine driven by its specific Na^+^-dependent co-transporter system can be applied to the modulation of D-serine.

There is also some discussion about the nature of the NMDA NR_2_ subunits on the inhibitory cell types in cortical networks. mRNA studies in the human brain localize the NR_2C_ subunit predominantly to the cerebellum (Monyer et al., 1992) although there are lower levels present in the cortex (Allen Brain institute data http://human.brain-map.org/). However, NR_2D_ subunits are likely present in cortical areas on excitatory-inhibitory synapses and could play a predominant role in the generation of the inhibitory tone. The exact interaction parameters between glycine and its co-agonist binding site on the NMDA receptor in the human brain are unknown, but the computer model suggests a range of interaction parameters that would correspond to an inverse U-shape dose-response. Although adding a greater contribution of NR_2D_ to the inhibitory tone will increase the EC_50_ of the e-i interaction, it will also decrease the Hill coefficient as compared to the interaction of glycine with the e-e synapses, which has been shown to be favorable for an inverse U-shape dose-response.

The model presented here does not address the other modulatory agents such as extracellular proteins, zinc, polyamines, and neurosteroids. All these molecules can influence the dynamics of glycine-mediated amplification of NMDA-currents and we assumed that these modulators do not change in schizophrenia. In principle, if new data become available suggesting a change in these neuromodulators as a consequence of schizophrenia pathology, detailed biochemical data could be incorporated in this platform to estimate their impact.

In summary, this report simulates the anticipated dose-response of glycine level modulation on an emergent property (BOLD-fMRI) of a computer-based neuronal circuit that has been calibrated against clinical outcomes for negative symptoms. It provides a physiological explanation for the appearance of an inverse U-shape dose-response based on a biologically constrained set of interaction parameters between glycine and the co-agonist site on different types of NMDA-NR_2_ subunits and the electrogenic character of the Gly/2Na/Cl co-transport system. A notable limitation is that this study deals with the effect of glycine modulation in the absence of any antipsychotic medication and therefore does not reflect the real clinical study design.

### Conflict of interest statement

All authors are employees of In Silico Biosciences. Hugo Geerts and Athan Spiros are the inventors on two patents related to Quantitative Systems Pharmacology.

## Abbreviations

ASL-fMRI: Arterial Spin Labeling functional Magnetic Resonance Imaging
BOLD-fMRI: Blood Oxygen Level Dependent functional Magnetic Resonance Imaging
CBF: Cerebral Blood flow
DA: Dopamine
dlPFC: dorso-lateral Prefrontal Cortex
EC_50_: concentration at which 50% of maximal effect is reached
Enk: encephalin
ER: endoplasmic reticulum
GlyT1: Glycine Transporter subtype 1
mAChR: muscarinic acetylcholine receptor
mGluR: metabotropic glutamate receptor
MSN: Medium Spiny Neuron (major cell type in striatum)
NMDA: N-methyl-D-aspartate (glutamate receptor subtype)
PANSS: Positive and Negative Symptoms Scale in schizophrenia
PDSP: Psycho-active Drug Screening Program
PET: Positron Emission Tomography
PFC: Prefrontal Cortex
PSD-95: postsynaptic density complex
QSP: Quantitative Systems Pharmacology
SP: Substance P
vmPFC: ventro-medial Prefrontal Cortex.
